# Event and Comment

**Published:** 1918-07

**Authors:** 


### DENTAL REGISTER

Vol. LXXII JULY, 1918 No. 7

### EVENT AND COMMENT

**Dentistry and the War** The announcement comes from Washington that no more dentists will be commissioned for professional services, as enough are now enlisted to care for an army of five million soldiers. This results from the inclusion of so many dentists in the draft and because of the large early enlistment of dentists not subject to draft.

The fact that nearly six thousand dentists are now enlisted is a gratifying comment on the loyalty of the profession. In addition a large proportion of the present graduating classes will be taken in the next call. This, however, will not materially augment the number of dentists available for commissioned service, as practically all have been already counted as they have been in the Reserve Corps for the past year. All of these men will, however, be lost to the profession, and six thousand dentists taken from the total practitioners of the country is bound to interfere considerably with the needs of our civil population. Perhaps we shall not need so much dentistry because of our more careful observance of oral hygiene principles, and the fact that we shall be forced to live on a simpler and more wholesome diet.

At any rate, we are in for the war and we must use every available means for conserving the health of our teeth as well as our food and materials needed to win the war. The dentists who remain at home will need to work all the harder to keep the dental equilibrium as nearly up to the average as possible. Our duty seems clearly outlined.

**The Preparedness League**

The reports from the president of the League indicate that this organization is meeting its problems in a most commendable fashion. It is expected that it will be able to report at the National meeting a half million operations made by the profession to fit our soldiers for service. An urgent call is made for all dentists to enroll in this service and to be sure to report all operations through the State Director, or through the headquarters at 50 E. Forty-Second Street, New York City.

The League has prepared a special course of instruction for dentists who may want to prepare themselves to engage in repair dentistry for soldiers who are returning from the war with serious dental injuries. President Beach, 131 Allen Street, Buffalo, will be glad to give full information in regard to this course. The League has prepared lantern slides giving information to the public and to dental societies as to the work of the League, that may be had on request to the President.

Major Heckard, who has given so much valuable effort to organize the League propaganda, has resigned, to take up special work in the Officers' Training Camp, in Camp Greenleaf, where he hopes to take over some very important service in addition to keeping up his interest and efforts in behalf of the League.

There are undoubtedly many dentists who are giving free service in preparing men for enlistment who have not yet joined the League or who do not report these services. It is desirable that every such dentist join the League and get the cards for reporting, that the work may be properly credited and recorded.

